# Focal Adhesion Kinase Inhibitors in Combination with Erlotinib Demonstrate Enhanced Anti-Tumor Activity in Non-Small Cell Lung Cancer

**DOI:** 10.1371/journal.pone.0150567

**Published:** 2016-03-10

**Authors:** Grant A. Howe, Bin Xiao, Huijun Zhao, Khalid N. Al-Zahrani, Mohamed S. Hasim, James Villeneuve, Harmanjatinder S. Sekhon, Glenwood D. Goss, Luc A. Sabourin, Jim Dimitroulakos, Christina L. Addison

**Affiliations:** 1 Cancer Therapeutics Program, Ottawa Hospital Research Institute, Ottawa, ON, Canada; 2 Department of Cellular and Molecular Medicine, University of Ottawa, Ottawa, ON, Canada; 3 Department of Biochemistry, Microbiology and Immunology, University of Ottawa, Ottawa, ON, Canada; 4 Department of Surgery, University of Ottawa, Ottawa, ON, Canada; 5 Department of Pathology and Laboratory Medicine, University of Ottawa, Ottawa, ON, Canada; 6 Department of Medicine, University of Ottawa, Ottawa, ON, Canada; Institute of Biomedical Sciences, TAIWAN

## Abstract

Blockade of epidermal growth factor receptor (EGFR) activity has been a primary therapeutic target for non-small cell lung cancers (NSCLC). As patients with wild-type EGFR have demonstrated only modest benefit from EGFR tyrosine kinase inhibitors (TKIs), there is a need for additional therapeutic approaches in patients with wild-type EGFR. As a key component of downstream integrin signalling and known receptor cross-talk with EGFR, we hypothesized that targeting focal adhesion kinase (FAK) activity, which has also been shown to correlate with aggressive stage in NSCLC, would lead to enhanced activity of EGFR TKIs. As such, EGFR TKI-resistant NSCLC cells (A549, H1299, H1975) were treated with the EGFR TKI erlotinib and FAK inhibitors (PF-573,228 or PF-562,271) both as single agents and in combination. We determined cell viability, apoptosis and 3-dimensional growth *in vitro* and assessed tumor growth *in vivo*. Treatment of EGFR TKI-resistant NSCLC cells with FAK inhibitor alone effectively inhibited cell viability in all cell lines tested; however, its use in combination with the EGFR TKI erlotinib was more effective at reducing cell viability than either treatment alone when tested in both 2- and 3-dimensional assays *in vitro*, with enhanced benefit seen in A549 cells. This increased efficacy may be due in part to the observed inhibition of Akt phosphorylation when the drugs were used in combination, where again A549 cells demonstrated the most inhibition following treatment with the drug combination. Combining erlotinib with FAK inhibitor was also potent *in vivo* as evidenced by reduced tumor growth in the A549 mouse xenograft model. We further ascertained that the enhanced sensitivity was irrespective of the LKB1 mutational status. In summary, we demonstrate the effectiveness of combining erlotinib and FAK inhibitors for use in known EGFR wild-type, EGFR TKI resistant cells, with the potential that a subset of cell types, which includes A549, could be particularly sensitive to this combination treatment. As such, further evaluation of this combination therapy is warranted and could prove to be an effective therapeutic approach for patients with inherent EGFR TKI-resistant NSCLC.

## Introduction

Lung cancers account for more deaths worldwide than any other type of cancer [1] with ~80% of lung cancers being classified as non-small cell lung cancers (NSCLC) [2]. The epidermal growth factor receptor (EGFR) protein is over-expressed in up to 80% of NSCLCs, hence EGFR has been a primary therapeutic target for NSCLC [3,4]. To this end, agents have been designed to target both the extracellular domain and intracellular kinase domain of EGFR. Inhibitors targeting the kinase domain of EGFR, such as erlotinib and gefitinib, have shown promise in patients with activating mutations (i.e. in exons 18, 19 or 21) in EGFR [5–8], although these inhibitors have demonstrated only modest benefits for patients harboring wild-type EGFR [9,10]. Additionally, secondary mutations in EGFR or c-MET amplification can develop, conferring resistance in previously sensitive patients [11]. As the incidence of EGFR activating mutations is relatively low in the majority of North American and European populations [12–15], there is a need to enhance the sensitivity to EGFR tyrosine kinase inhibitors (TKIs) for patients with wild-type EGFR.

Focal adhesion kinase (FAK) is a non-receptor tyrosine kinase that localizes at sites of cell adhesion to the extracellular matrix (ECM) and mediates signalling events downstream of integrin engagement of the ECM. FAK is known to regulate cell survival, proliferation and migration [16]. FAK expression has also been shown to be up-regulated in many cancer types including lung cancers [17], thus positioning FAK as an important target for regulation in cancer therapy. To this end, FAK inhibitors have been developed, including pharmacological inhibitors of FAK tyrosine kinase activity [18,19]. Inhibition of FAK has been demonstrated to affect a number of cellular processes important for tumor growth and disease progression including angiogenesis and metastasis [20–22]. Additionally, FAK inhibitors have been shown to effectively inhibit tumor growth in a number of subcutaneous xenograft models [23,24] showing promise as single agents as well as in combination with other inhibitors [24–26].

In NSCLC, increased expression levels of FAK are observed in tumor tissue as compared to normal lung tissue, and this increased expression is correlated with higher disease stages [27]. These findings suggest an important role for FAK in the progression of NSCLC. Recent evidence has also implicated β1 integrin expression in resistance to the EGFR TKI gefitinib, with increased gefitinib sensitivity being seen following β1 integrin depletion in NSCLC cells [28]. Given that FAK is one of the main kinases activated downstream of β1 integrin, the importance of ECM-focal adhesion complex signalling in resistance to EGFR TKI treatment is indicated. As it is an established practice to treat NSCLC patients with EGFR TKIs and there increasing evidence that FAK plays a major role in lung cancer growth and progression, we set out to test the utility of combining the EGFR inhibitor erlotinib with FAK inhibition in NSCLC. We investigated the effects of two FAK inhibitors, PF-573,228 (PF-228) and PF-562,271 (PF-271) on NSCLC cell growth in culture and tumor growth in mouse xenograft models as both single agents and in combination with erlotinib. The results of our study indicate that combining FAK inhibition with erlotinib more effectively reduces EGFR wild-type NSCLC cell viability *in vitro* and xenograft tumor growth *in vivo* than either drug treatment alone, with particular efficacy in the A549 cell type. Thus, our results have identified a promising drug combination strategy targeting EGFR and FAK in NSCLC, and indicate that a treatment regimen including a FAK inhibitor may prove more beneficial than treatment with erlotinib alone in patients harboring inherent EGFR TKI-resistant NSCLC.

## Methods

### Cell culture and reagents

Human cell lines A549 (EGFR wild-type) and H1299 (EGFR wild-type) were purchased from ATCC, while H1975 (EGFR L858R and T790M mutations), HCC827 (EGFR ΔE746-E750) and HCC4006 (EGFR ΔL747-E749) cell lines were a gift of Dr. Ming Tsao (Princess Margaret Hospital, Toronto, ON). A549 cells were maintained in DMEM (Mediatech, Manassas, VA) supplemented with 10% fetal bovine serum (FBS; Medicorp, Montreal, QC), while H1299, H1975, HCC827 and HCC4006 cells were maintained in RPMI-1640 (HyClone Laboratories, Logan, UT) with 10% FBS. All cell lines were grown at 37°C and 5% CO_2_. The FAK inhibitor PF-573,228 (PF-228) was purchased from Tocris Bioscience (Bristol, UK) and dissolved in DMSO. Erlotinib and the FAK inhibitor PF-562,271 (PF-271) were purchased from Shanghai Biochempartner Co. Ltd. (Shanghai, China) and dissolved in DMSO for *in vitro* cell culture work.

### Generation of erlotinib resistant cells

HCC4006 cells which were originally sensitive to erlotinib, were made resistant to erlotinib through 5 rounds of dose escalation starting with 0.005 μM erlotinib and increasing concentration by a factor of 5 until cells resistant to 3 μM erlotinib were isolated. Parental cells were treated with equivalent amounts of DMSO to cells undergoing selection for resistance for the duration of the dose escalation experiment, and these were used as controls for all subsequent experiments.

### Plasmid transfection of LKB1 into A549 cells

A549 cells were transfected with pcDNA3-FLAG-LKB1 (plasmid 8590 [29], Addgene, Cambridge MA) to overexpress LKB1 or with pcDNA3.1 as vector control using GeneJuice transfection reagent (Novagen, Etobicoke, ON). Transfected cells were seeded into multiple tissue culture dishes and after 48 hours, actively dividing cells were treated with 500 μg/ml Geneticin (Invitrogen, Burlington, ON) to facilitate selection of plasmid-expressing cells. Cells in each tissue culture dish remaining after selection were pooled to create a number of pooled clones of both LKB1 expressing and non-expressing A549 cells. Confirmation of LKB1 expression in FLAG-LKB1 transfected cells and continued lack of LKB1 expression in control cells was confirmed by western blotting for LKB1.

### siRNA transfection

H1299 cells were either mock transfected (PBS) or transfected with siGENOME non-targeting control siRNA #2 or siGENOME SMARTpool LKB1 siRNA (cat #M-005035-02, Dharmacon, Lafayette, CO) with Oligofectamine reagent (Invitrogen, Burlington, ON). Efficiency of LKB1 knockdown was confirmed through western blotting. For MTT assays utilizing siRNA-transfected cells, drug treatment commenced at 48 hours post transfection. For experiments involving stimulation of cells with EGF for western blotting, siRNA-transfected cells were serum starved overnight beginning at 48 hours post transfection prior to growth factor stimulation.

### MTT cell viability assay

Cells were seeded in 96-well plates at a density of 4,000 cells/well and incubated overnight. For dose escalation and drug combination experiments, cells were treated with either erlotinib or PF-228 alone or in combination at various concentrations of drug in DMEM or RPMI-1640 supplemented with 5% FBS for 48 hours. Cell viability was then assessed with MTT reagent (Thiazolyl blue tetrazolium bromide; Sigma, Oakville, ON) as previously described [30] with absorbance being determined at 570 nm with a Multiskan Ascent plate reader (Thermo Scientific, Rockford, IL). Treatments were performed in replicates of six with the mean values expressed as the percent viability relative to the DMSO treated control (100% viable). The nature of the effect of erlotinib in combination with the FAK inhibitor PF-228 was determined by the Chou-Talalay method [31] using CalcuSyn software (Biosoft, Cambridge, UK). Data for cell viability as measured by MTT for each drug alone and in combination was used in the CalcuSyn program to produce fraction-affected CI (Fa-CI) plots indicating the combination index (CI) values for each drug combination. CI values < 1 indicate synergistic interactions, CI values > 1 indicate antagonistic interactions and CI values = 1 indicate additive interactions.

### FAK immunoprecipitation and in vitro kinase assay

Immunoprecipitation and kinase reactions were performed as previously described [32]. Briefly, equal amounts of total cell lysate (600 μg) were immunoprecipitated for FAK with anti-FAK antibody (BD Bioscience, Mississauga, ON) and 20 μl of Protein A/G sepharose beads (GE Healthcare, Uppala, Sweden) while rotating for 2 hours at 4°C. The beads were then washed 3 times with 200 mM NETN (20 mM Tris-HCl, pH 8.0, 1 mM EDTA, pH 8.0, 200 mM NaCl, 0.5% Igepal CA-630) followed by one wash in kinase buffer (0.25 mM NaVO_3_, 20 mM Tris-HCl, pH 7.4, 1 mM NaF, 10 mM β-glycerophosphate, 1 mM dithiothreitol, 15 mM MgCl_2_). DMSO (vehicle control) or FAK inhibitor PF-228 at 1 or 5 μM concentrations were then added to the reaction in 20 μl of kinase buffer and incubated for 20 minutes at 30°C. The kinase assay was then initiated with 1 μl of [^32^P] γATP (5 μCi/μl) and incubated for 30 minutes at 30°C with mixing every 10 minutes. The reaction was terminated following the addition of 4X SDS sample buffer. The samples were resolved on a 7.5% polyacrylamide gel and transferred to PVDF membrane. The membrane was subjected to autoradiography for development of FAK phosphorylation events and probed for total FAK levels by western blotting as described below.

### Flow cytometry

Cells were treated with solvent control (DMSO), erlotinib, PF-228, or both erlotinib and PF-228 in medium containing 5% FBS for 48 hours. Both non-adherent and adherent cells were collected and pooled, washed twice with PBS and resuspended in 70% ethanol for permeabilization. Cell suspensions were then treated with propidium iodide solution (48 μg/ml propidium iodide, 40 μg/ml RNase A) for 30 minutes at room temperature. A total of 1 x 10^4^ cells were evaluated using the Beckman Coulter Epics XL flow cytometer (Beckman-Coulter, Mississauga, ON) and the percentage of apoptotic cells was calculated from cells with less than 2N DNA content using FCS Express flow cytometry analysis software (De Novo Software, Los Angeles, CA).

### Western blot analysis

Following total protein extraction, western blotting was performed as previously described [30]. Primary antibodies used in this study were as follows: rabbit anti-Akt (Cat. # 9272), pAkt (S473, Cat. # 9271), LKB1 (Cat. # D60C5) and PARP (Cat. # 9542) antibodies were from Cell Signaling Technology (Danvers, MA), mouse anti-FAK (clone 77/FAK) was from BD Transduction Laboratories (Mississauga, ON), and mouse anti-β-actin antibody (clone AC-74) was from Sigma (St. Louis, MO). Following incubation with primary antibody overnight, membranes were washed 3 x 5 minutes and incubated in horse radish peroxidase (HRP) conjugated goat anti-mouse or goat anti-rabbit secondary antibody (Calbiochem, San Diego, CA) for 1 hour. Membranes were then washed 6 x 5 minutes and incubated in Immobilon Western Chemiluminescent HRP Substrate (EMD Millipore, Billerica, MA) prior to image development using the GeneGnome Bio Imaging System and GeneSnap software (Syngene, Frederick, MD).

### Colony growth assay

Lab-Tek II 4-well chamber slides (Nalge Nunc International, Naperville, IL) were coated with 100 μl of reduced growth factor basement membrane extract (BME; Trevigen, Gaithersburg, MD) which solidified for 30 minutes at 37°C. Cells were then seeded onto the solidified BME layer at a density of 5,000 cells/well in medium containing 10% FBS and 2.5% BME. Medium was refreshed every three days. Cell colony sizes were determined with ImageJ software from a total of 4 images from each of duplicate wells for each experiment.

### In vivo tumor growth

A549 lung cancer cells (1 x 10^6^ cells) were injected subcutaneously into both the left and right hind flanks of 6-week old CD-1 nude mice (Charles River, Wilmington, MA). Mice were housed in specific pathogen free conditions, on a 12 hour light/dark cycle with ad libitum feeding and water for the duration of the experiment. Three days after injection, mice (4 mice/treatment) were first treated by oral gavage with vehicle control, or with previously demonstrated effective concentrations of erlotinib (50 μg/g) [33], PF-271 (50 μg/g) [23], or the combination of both drugs in PBS with 5% gelucire (vehicle control). Drugs were then administered by oral gavage daily for five consecutive days followed by two days without treatment and the process was then repeated for the duration of the experiment. Measurements of tumor size were taken once a week by caliper measurement and tumor volume was calculated using the following formula: tumor volume = (width)^2^ x (length)/2. All animal experiments were performed and approved by the University of Ottawa Animal Care Committee and conformed to the guidelines set by the *Animals for Research Act* and the Canadian Council on Animal Care's *Guide to the Care and Use of Experimental Animals* (Vol. 1, 2nd ed., 1993), and meet standards of practice in the disciplines of laboratory animal science and laboratory animal veterinary medicine. As per approved protocol, animals were euthanized by carbon dioxide asphyxiation followed by cervical dislocation at pre-determined experimental endpoints including a) tumor mass to the point where it significantly interferes with normal bodily functions or causes pain, b) Consistent or rapid body weight loss exceeding 20% of the body weight c) ulceration/infection of the tumor site (breakage of skin barrier), e) Tumor burden >10% of mouse’s body weight g) Severe respiratory distress or h) ambulatory distress for any reason.

### Ex vivo lung tumor analysis

Surgically resected NSCLC tumors and normal adjacent lung tissue were collected after pathological evaluation from five patients who provided written informed consent as per protocol approved by The Ottawa Hospital Research Ethics Board (Protocol # 20120559-01H). Patient characteristics are described in Table 1. Tissue was processed the same day for use in *ex vivo* experiments. A biopsy punch (Miltex GmbH, Rietheim-Weilheim, Germany) was used to obtain 2 mm diameter cores from both lung tumor tissue and normal lung tissue and further sectioned into 1 mm thick slices with three slices then randomly distributed into triplicate wells of a 24-well tissue culture plate. Tissue slices were maintained in DMEM supplemented with 10% FBS and 100 U/ml antibiotic/antimycotic solution (Gibco, Waltham, MA) at 37°C and 5% CO_2_. As each tissue slice is not necessarily identical in terms of its cell density or viability at the time of isolation, the day following isolation, viability of tissue slices was assessed on a per well basis prior to drug addition following incubation for 4 hours with a 10% alamarBlue solution (AbD Serotec, Raleigh, NC). Post drug treatment readings were then normalized to these baseline readings of viability for each well. Tissue slices were treated the day following isolation with either vehicle control (DMSO), erlotinib (10 μM), PF-271 (5 μM), or the combination of both drugs for 48 hours and cell viability was then assessed by 10% alamarBlue solution. Fluorescence was measured (excitation 530 nm/emission 590 nm) with a Fluoroskan Ascent fluorescence plate reader (Thermo Scientific, Rockford, IL) 4 hours post addition of alamarBlue.

**Table 1:** Patient characteristics.

Sex	Age	Stage	Nodal Status	Tumor Type
Male	61	pT2a	N1	Adenocarcinoma
Male	63	pT2a	N0	Adenocarcinoma
Female	74	pT2a	N0	Adenocarcinoma
Male	55	pT4	N1	Adenocarcinoma
Male	81	pT2a	N0	Squamous

### Statistical analyses

All statistical analyses were performed using Prism 6.0 (GraphPad, La Jolla, CA). Multiple comparisons were performed by ANOVA whereas single comparisons were performed by unpaired Student’s *t* tests. Statistically significant differences were determined as *P* < 0.05 from a minimum of two independently performed experiments.

## Results

### Inhibition of FAK reduces cell viability in EGFR TKI-resistant NSCLC cells

In order to assess the effectiveness of treating EGFR TKI-resistant NSCLC cell lines with FAK TKIs, we utilized two EGFR wild-type cell lines (A549, H1299) with known insensitivity to EGFR TKIs [34], as well as the EGFR mutant cell line H1975, with acquired EGFR TKI resistance due to a T790M mutation in the EGFR gene [35]. These three cell lines thus provided us with reasonable examples to test the effectiveness of adding a FAK tyrosine kinase inhibitor to erlotinib treatment in order to more potently inhibit the growth of EGFR TKI-resistant NSCLC cells.

**Fig 1:**
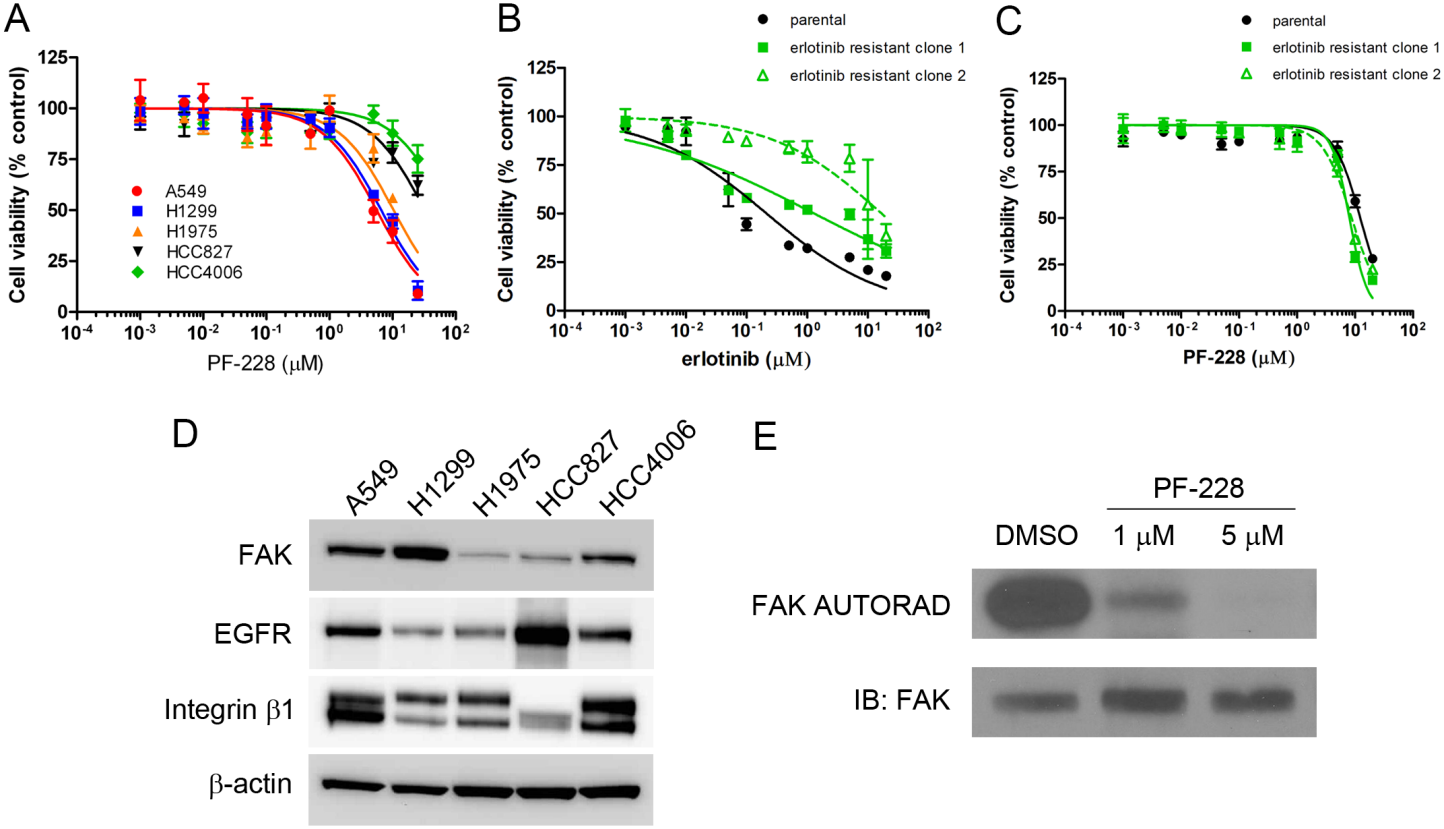
Inhibition of FAK reduces cell viability of EGFR TKI-resistant NSCLC cells. (**A**) Cell viability was assessed following treatment with PF-228 at varying doses for 48 hours in complete media with 5% FBS. Cell viability was assessed by MTT assay with the viability of DMSO-treated cells set as 100%. Data indicates the mean ± SEM for log[inhibitor] vs. normalized response from two independently performed experiments. (**B**) HCC4006 cells serially grown in increasing concentrations of erlotinib up to 3 μM were confirmed resistant compared to parental cells following MTT assay of cells treated with increasing concentrations of erlotinib. Data is graphed as the mean of log[inhibitor] vs normalized response ± SEM (N = 2). (**C**) Erlotinib resistant HCC4006 cells are more sensitive to FAK inhibitor PF-228 than parental HCC4006 cells. Cells were treated with increasing concentrations of PF-228 and cell viability was assessed by MTT assay. Data is presented as the mean of log[inhibitor] vs normalized response ± SEM (N = 2). (**D**) Relative protein expression between erlotinib resistant (A549, H1299, H1975) and sensitive (HCC827, HCC4006) parental cell lines. (**E**) A549 cell lysates were immunoprecipitated for endogenous FAK and subjected to *in vitro* kinase assays in the presence of either DMSO (vehicle control) or PF-228 (1 μM or 5 μM). Autoradiography revealed FAK phosphorylation (FAK AUTORAD) and western blotting indicated total levels of FAK (IB: FAK).

We first tested the FAK inhibitor PF-228 [36] and assessed its ability to dose-dependently (from 0.001 to 25 μM) inhibit the growth of NSCLC cell lines *in vitro*. Treatment of cells with escalating doses of PF-228 indicated that all three EGFR TKI-resistant cell lines (A549, H1299, H1975) were sensitive to this drug at concentrations greater than 1 μM (Fig 1A). IC50 values for PF-228 determined from the graph were: 5.6 μM (R^2^ = 0.92) in A549, 6.6 μM (R^2^ = 0.95) in H1299 and 10.4 μM (R^2^ = 0.80) in H1975. Interestingly, viability of two EGFR TKI-sensitive cell lines (HCC827, HCC4006) [34] was much higher (~ 75% of cells remaining viable compared to control treated) than the EGFR TKI-resistant cell lines (~ 10% of cells remaining viable compared to control treated) at the highest dose of PF-228 tested (25 μM) (Fig 1A), although the reason for this difference in viability is not yet understood. As the two cell lines which had inherent resistance to erlotinib and were both EGFR wild-type appeared to be more sensitive to the FAK inhibitor than the H1975 cells which had EGFR mutations resulting in acquired sensitivity, we examined whether cells initially sensitive to erlotinib also became more sensitive to FAK upon gaining acquired drug resistance. HCC4006 cells which were rendered resistant to erlotinib following serial passaging in increasing concentrations of erlotinib up to 3μM, resulted in cell clones with significantly less sensitivity to erlotinib with IC50s of 1.3 μM (R^2^ = 0.87) and 16.8 μM (R^2^ = 0.73) for resistant clones 1 and 2 respectively as compared to control cells that were serially passaged in equal volumes of DMSO control with an IC50 of 0.21 μM (R^2^ = 0.91) (Fig 1B). It should be noted that both of these clones exhibited erlotinib resistance at similar or higher levels to the resistant parental lines chosen (with IC50s ranging from 1.2μM, 5.3μM and 5.9μM for A549, H1299 and H1975 respectively). Erlotinib resistant HCC4006 cells showed increased sensitivity to FAK inhibitors with IC50s of 8.0 μM (R^2^ = 0.96) and 8.7 μM (R^2^ = 0.95) for clones 1 and 2 respectively compared to control HCC4006 clone with an IC50 of 12.3 μM (R^2^ = 0.90) (Fig 1C). While erlotinib resistant cell clones did show increased sensitivity to FAK inhibitors, they remained less sensitive to PF-228 as compared to EGFR wild-type inherently erlotinib resistant A549 or H1299 cells. It is thus unlikely that the main mechanism of acquired resistance to erlotinib in EGFR mutant NSCLC depends significantly on FAK activity.

We also evaluated whether the sensitivity to FAK inhibitors observed in the parental NSCLC cell lines was associated with basal expression of any major proteins in these drug target pathways. The sensitivity to FAK drug did not appear to correlate with overall levels of FAK, EGFR or β1 integrin proteins, as these levels appeared variable in both sensitive and insensitive cell lines (Fig 1D). Given that the three EGFR TKI-resistant NSCLC cell lines had IC50 values for PF-228 ranging from 5 to 10 μM, and that our lab had previously observed inhibition of both autophosphorylation and kinase activity of FAK at 5 μM PF-228 [20], we confirmed the activity of this inhibitor in A549 cells following *in vitro* kinase assays for FAK autophosphorylation. The addition of PF-228 to the kinase reaction significantly inhibited FAK autophosphorylation in A549 cells (Fig 1E) with similar results being observed in H1299 and H1975 cell lines (data not shown). Thus, subsequent experiments were performed using concentrations of PF-228 in the range of 1–10 μM.

### FAK inhibitor PF-228 synergizes with erlotinib to more effectively reduce cell viability in EGFR wild-type NSCLC cells

**Fig 2:**
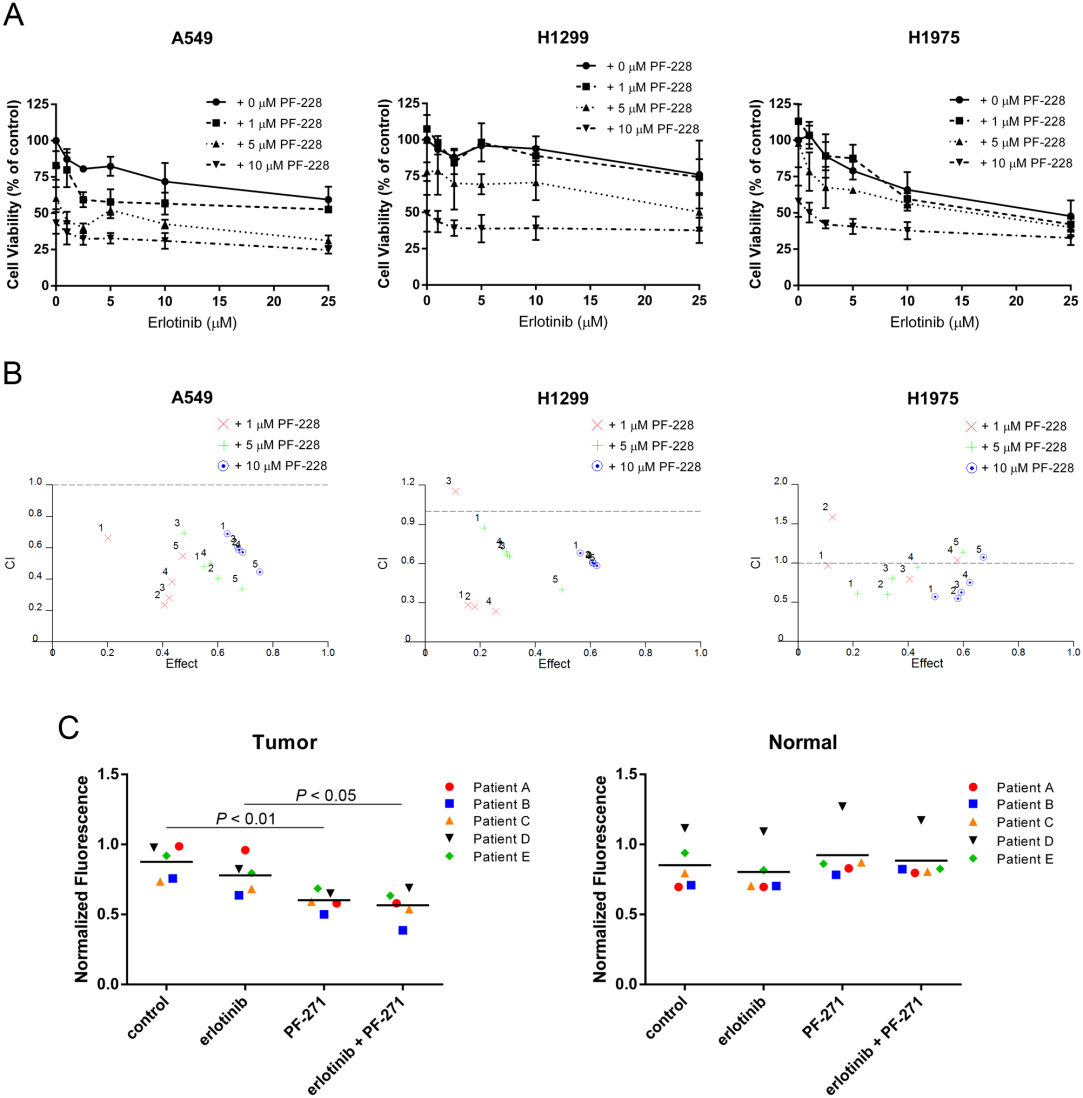
Erlotinib cytotoxicity is synergistically enhanced by the FAK inhibitor PF-228. (**A**) Cells were treated with PF-228 and erlotinib for 48 hours and assayed for cell viability using MTT assay. Data indicates the mean ± SEM for cell viability presented as a percentage of control DMSO-treated cells (100% viable) from two independently performed experiments. (**B**) Fraction affected/Combination index (CI) plots indicating the synergistic cytotoxicity of erlotinib and PF-228. CIs < 1 are considered synergistic, CIs > 1 are considered antagonistic, and CIs = 1 are considered additive. (**C**) FAK inhibition reduces cell viability in human lung tumor tissue but not normal lung tissue *ex vivo*. Tissues from five unselected patients were treated for 48 hours with vehicle control (DMSO), erlotinib (10 μM), PF-271 (5 μM), or the combination of erlotinib and PF-271. Cell viability was assessed for both tumor tissue and normal tissue by alamarBlue from three wells per condition with each well containing three 2 mm x 1 mm tissue pieces. Fluorescence was normalized to baseline fluorescence readings for each well taken prior to drug treatment. Experimentation was performed independently on each patient sample Mean fluorescence is indicated for each condition as a horizontal line and statistically significant differences were determined by ANOVA and indicated as *P* values above the comparison.

As treatment with FAK TKI alone appeared to inhibit the viability of the EGFR TKI-resistant NSCLC cells tested in Fig 1, we next wanted to determine whether addition of PF-228 could sensitize cells to erlotinib, or at the very least, result in an additive reduction in cell viability above and beyond that of treatment with erlotinib alone. Cells were thus treated with erlotinib (1–25 μM) or PF-228 (1–10 μM) alone and in combination and cell viability was assessed by MTT activity. All three cell lines tested exhibited resistance to erlotinib treatment alone even at very high concentrations (i.e. 25 μM, Fig 2A) confirming the results of previous studies regarding EGFR TKI resistance in both EGFR wild-type and T790M mutation-containing cell lines [34,35,37–39]. When erlotinib and PF-228 were used in combination we observed an increased cytotoxic effect in A549, H1299 and H1975 cells as we had hypothesized (Fig 2A). The Chou-Talalay method was used to determine the nature of the drug combination effect [31]. The combination index (CI) was determined through plotting the fraction affected where CI values less than 1 are considered synergistic, CI values greater than 1 are considered antagonistic, and CI values equal to 1 are considered additive. The combination of erlotinib and PF-228 resulting in reduced cell viability was found to be synergistic in all three cell lines tested (Fig 2B). As a secondary system we employed the use of human lung cancer patient tumor explants to further test the effectiveness of FAK TKIs in lung cancer cells. Tumor explants from unselected patients undergoing thoracotomy for early stage lung cancers were treated *in vitro* with erlotinib or FAK TKIs alone or in combination. The treatment of explanted lung tumor with erlotinib alone showed a modest but not statistically significant decrease in cell viability (Fig 2C, left panel). Similar modest reductions of cell viability were also observed in adjacent normal lung tissues following erlotinib treatment alone (Fig 2C, right panel). We observed that patient lung cancers were also sensitive to FAK TKI inhibition with a trend towards more effective inhibition when used in combination with erlotinib (Fig 2C). Despite the small sample size and variability associated with testing in heterogeneous tissues (i.e. different ratios of tumor to stroma may occur in each patient sample), we were encouraged by the obvious benefit of the drug combination over erlotinib treatment alone, similar to what we observed in our cell line experiments. Of additional importance, adjacent normal lung tissue appeared to be unaffected by FAK TKI treatments alone or in combination with erlotinib (Fig 2C), suggesting a potential selective inhibition of NSCLC tumors.

**Fig 3:**
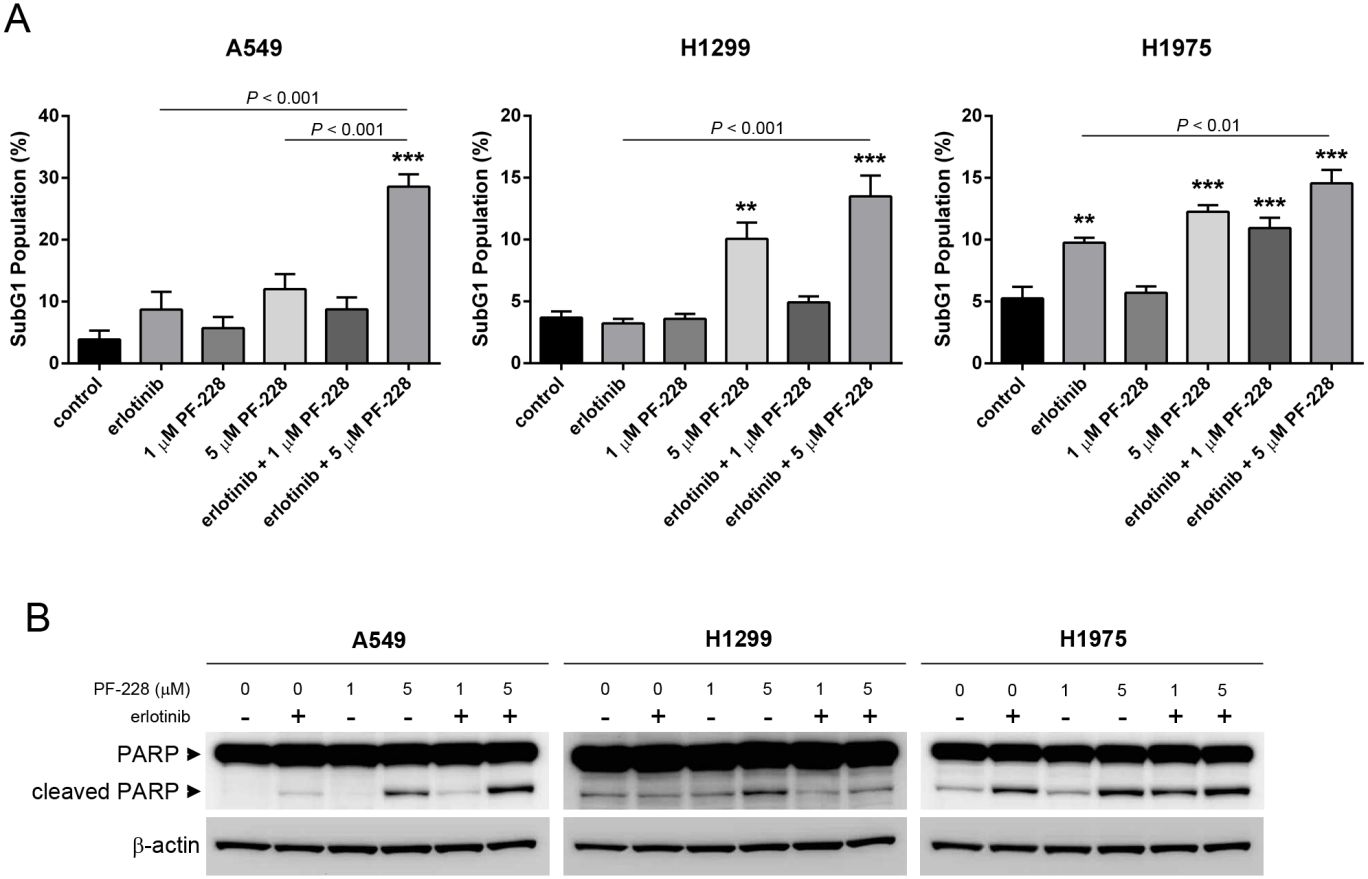
Increased apoptosis in NSCLC cells treated with erlotinib and FAK inhibitor PF-228 in combination. (**A**) Apoptotic cell fraction as determined by the percentage of cells in SubG1 cell cycle phase using propidium iodide staining and flow cytometric analysis following treatment for 48 hours with either vehicle control (DMSO), erlotinib (10 μM), PF-228 (1 μM or 5 μM), or both erlotinib and PF-228. Data presented is the mean ± SEM from two independently performed experiments. Statistically significant differences were determined by ANOVA. Asterisks denote statistically significant differences compared to control treated cells (** *P* < 0.01, *** *P* < 0.001), while significant differences between other treatment groups are displayed as *P* values above the comparison. (**B**) Western blot analysis indicating increased levels of cleaved PARP following drug treatment for 48 hours. Cells were treated with either vehicle control (DMSO), erlotinib (10 μM), PF-228 (1 μM or 5 μM), or both erlotinib and PF-228. β-actin was used as loading control. Data presented is representative of results obtained from two independently performed experiments.

In order to determine if the observed decrease in cell viability correlated with an increased level of apoptosis in treated cells, we analyzed the SubG1 population of cells following treatment with either erlotinib or PF-228 alone or in combination using propidium iodide staining of DNA content and flow cytometry analysis. In A549 cells, the combination treatment of erlotinib and PF-228 (5 μM treatment) resulted in a statistically significant increase in the SubG1 population of cells compared to control (~ 6-fold increase), in addition to exhibiting a significant increase in the percentage of SubG1 cells compared to both the erlotinib treatment alone (*P* < 0.001) and PF-228 treatment alone (*P* < 0.001) (Fig 3A). Increased levels of cleaved PARP in A549 cells above the levels seen following treatment with either drug alone, were also observed in cells treated with the combination of erlotinib and PF-228 (Fig 3B), confirming increased apoptosis in these cells. Although treatment of both H1299 and H1975 cells with 5 μM PF-228 alone or in combination resulted in a statistically significant increase in the SubG1 population compared to vehicle control or erlotinib treatment alone (Fig 3A; *P* < 0.001 in H1299, *P* < 0.01 in H1975 compared to erlotinib), the combination treatments in these cell lines were not significantly better than PF-228 used as single agent, although a trend towards increased efficacy was observed in both cell lines. In contrast to A549 cells, observed levels of cleaved PARP following treatment with the drug combination did not differ dramatically from that seen following treatment with erlotinib or FAK TKI alone in H1299 and H1975 (Fig 3B), confirming a less potent effect of erlotinib and PF-228 combination treatment on apoptosis in these cell lines. Thus, these data indicate that treatment with FAK inhibitor PF-228 in combination with erlotinib more potently induces an apoptotic shift in cell population than treatment with erlotinib alone, and in A549 cells specifically, this shift is significantly greater than either drug treatment alone.

### Treatment with erlotinib and PF-228 inhibits phosphorylation of Akt

**Fig 4:**
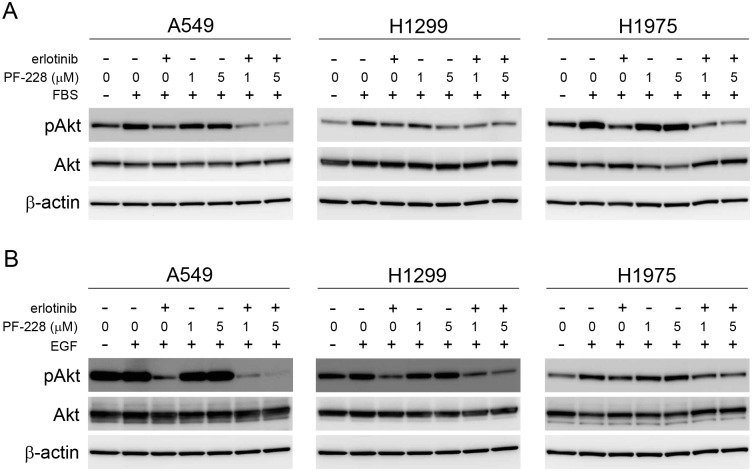
Akt phosphorylation is reduced following treatment with both erlotinib and PF-228. Serum starved cells were treated with either vehicle control (DMSO), erlotinib (10 μM), PF-228 (1 μM or 5 μM), or a combination of both erlotinib and PF-228 for 30 minutes prior to stimulation of cells with either (**A**) 5% FBS or (**B**) 100 ng/ml EGF for 30 minutes. Phosphorylated and total Akt were detected by western blotting. β-actin was used as loading control. Data presented is representative of results obtained from three independently performed experiments.

Recent evidence has indicated that β1 integrin expression negatively correlates with sensitivity to the EGFR TKI gefitinib in NSCLC cell lines, as β1 integrin depletion increased sensitivity to gefitinib resulting from enhanced inhibition of EGFR-induced phosphorylation of ERK and Akt [28]. As FAK can mediate signalling downstream of integrins, and persistent Akt activity has been indicated to contribute to a lack of response to EGFR TKIs [40], we assessed the status of Akt phosphorylation following treatment with erlotinib and PF-228 alone or in combination. In A549, H1299 and H1975 cells, treatment with erlotinib reduced both serum- (Fig 4A) and EGF-induced (Fig 4B) phosphorylation of Akt. Treatment with PF-228 alone did not affect serum- (Fig 4A) or EGF-stimulated (Fig 4B) phosphorylation of Akt in A549 and H1975 cells, while slight inhibition of Akt phosphorylation was observed in serum-stimulated H1299 cells (Fig 4A). Interestingly, treatment with both erlotinib and PF-228 reduced phosphorylation of Akt in response to serum (Fig 4A) or EGF (Fig 4B) to a greater extent than either treatment alone in A549 and H1975 cells, with A549 cells again showing the most potent inhibition. H1299 cells showed similar levels of phospho-Akt following treatment with the drug combination as compared to treatment with erlotinib alone (Fig 4). This suggests that the increased potency of the drug combination in A549 cells, may be due in part to enhanced inhibition of Akt phosphorylation which contributes to the decrease in cell viability and increased apoptosis seen when A549 cells are treated with a combination of erlotinib and PF-228.

### Enhanced efficacy of FAK TKI in combination with erlotinib does not appear to be dependent on LKB1

**Fig 5:**
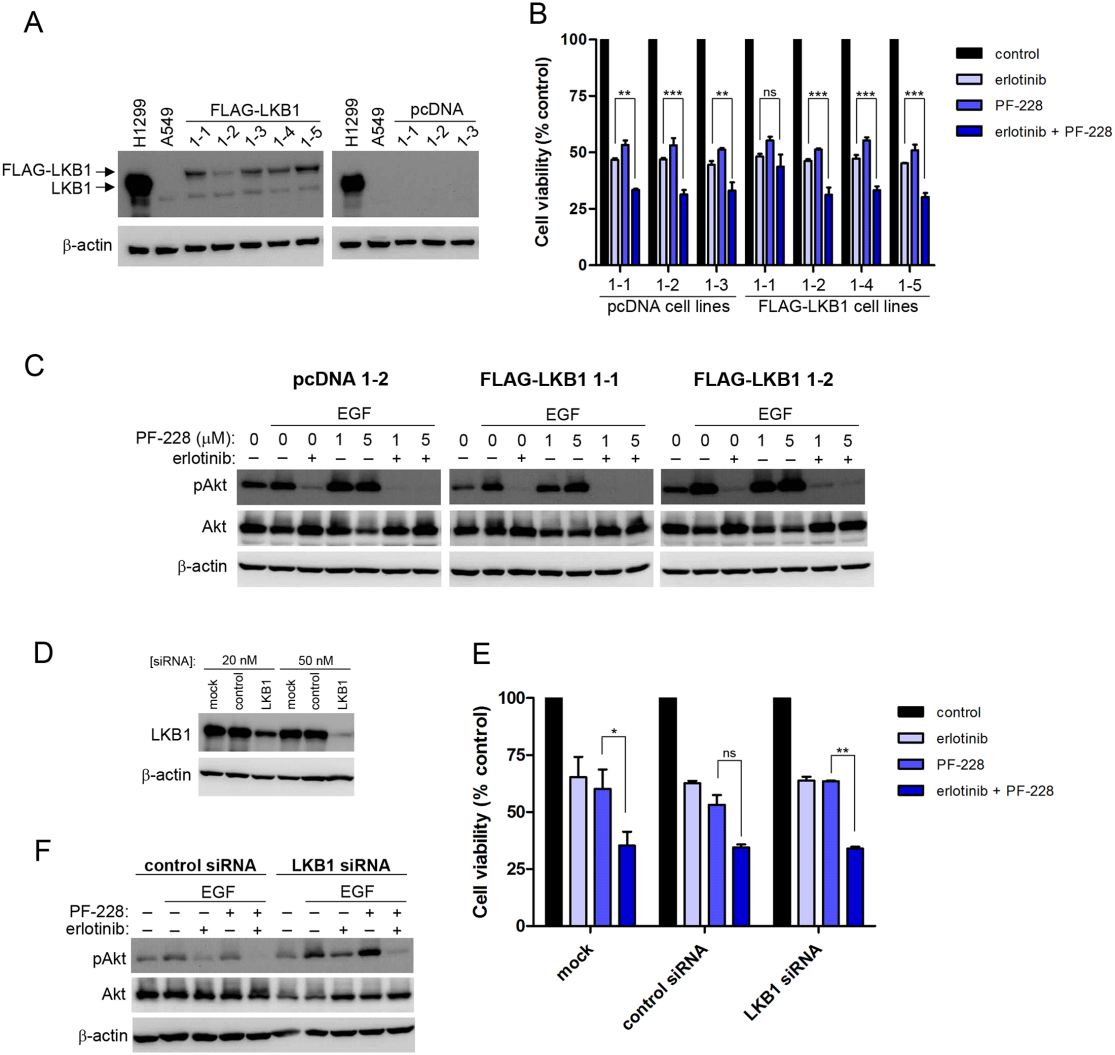
LKB1 does not appear to affect sensitivity to combination treatment with erlotinib and PF-228. (**A**) Confirmation of LKB1 expression in FLAG-LKB1 transfected A549 cells. Stably transfected cells were assessed for LKB1 expression by western blot. β-actin was used as loading control. H1299 cells normally express wild-type LKB1 and were used as positive control. (**B**) Expression of LKB1 in A549 cells does not alter sensitivity to erlotinib and PF-228. Cell viability was assessed by MTT assay following incubation with erlotinib (10 μM), PF-228 (5 μM) or both drugs for 48 hours. Data is presented as the mean ± SEM of two independently performed experiments. Statistically significant differences were determined by ANOVA (** *P* < 0.01, *** *P* < 0.001). (**C**) Expression of LKB1 in A549 cells does not alter the phosphorylation pattern of Akt following treatment with erlotinib and PF-228. Cells were serum-starved overnight and treated with erlotinib (10 μM) and/or PF-228 at the indicated concentrations for 30 minutes prior to stimulation with EGF (100 ng/ml) for 30 minutes. Levels of phospho- and total Akt were assessed by western blot. β-actin was used as loading control. (**D**) LKB1 is effectively depleted in H1299 cells following siRNA transfection. Expression of LKB1 was determined by western blot 48 hours post-siRNA transfection. β-actin levels were used as a loading control. Image is representative of two independently performed experiments. (**E**) Sensitivity of H1299 cells to erlotinib and PF-228 is unaltered following depletion of LKB1. H1299 cells were transfected with 50 nM of either control siRNA or LKB1 siRNA. Cell viability was assessed by MTT assay following a 48 hour treatment with erlotinib (10 μM), PF-228 (5 μM) or both drugs in combination. Data is presented as the mean ± SEM of two independently performed experiments. Statistically significant differences were determined by ANOVA (* *P* < 0.05, ** *P* < 0.01). (**F**) The phosphorylation pattern of Akt is unaltered in response to erlotinib and PF-228 in H1299 cells depleted of LKB1 as compared to control transfected cells. Cells were stimulated with EGF (100 ng/ml) following treatment with erlotinib (10 μM) and/or PF-228 (5 μM) for 30 minutes. Levels of phospho- and total Akt were assessed by western blot. β-actin was used as loading control. Image is representative of two independently performed experiments.

Given that we observed increased efficacy of the treatment combination in A549 cells with respect to the ability to block phospho-Akt and overall cell viability, we attempted to determine putative mechanisms which may be contributing to this observed increased efficacy. As A549 cells harbour a mutation in the LKB1/STK11 gene resulting in reduced LKB1 expression while H1975 and H1299 cells do not contain this mutation [41,42], we speculated that lack of LKB1 may be a contributing factor. This was further supported by published results demonstrating that LKB1 interacts with and inhibits FAK [43], and thus it is a possibility that A549 cells may be more sensitive to FAK inhibitors owing to an increased reliance on FAK signalling due to their lack of a LKB1-FAK inhibitory interaction. In order to investigate this possibility, we first transfected A549 cells with a plasmid expressing LKB1 and selected LKB1 overexpressing clones. Numerous pooled clones were generated and overexpression of LKB1 as compared to control plasmid transfected cell clones was confirmed by western blot analysis (Fig 5A). LKB1 expression in H1299 was included as a control. All selected clones appeared to express significantly higher levels of LKB1 as compared to both vector transduced and parental A549 cells. We next compared the sensitivity of the generated cell clones to erlotinib or PF-228 alone or in combination. In all cases regardless of LKB1 expression, all cell clones showed significant enhanced sensitivity to the combination of erlotinib and PF-228 as compared to treatment with either drug alone (Fig 5B). In a similar manner, ability to inhibit activation of Akt following EGF-stimulation was most significant when cells were treated with erlotinib in combination with PF-228 (Fig 5C) as was seen previously in parental A549 cells (Fig 4B). To confirm our findings, we also depleted LKB1 expression in H1299 cells which do not harbour a mutation in this gene. A pool of 4 independent siRNA targeting LKB1 were transfected into H1299, and a resulting dose dependent reduction in LKB1 protein was observed (Fig 5D). We then compared the sensitivity of siRNA transfected H1299 to erlotinib or PF-228 alone or in combination. A similar trend was observed whereby cells were more readily inhibited by the combination drug treatment as compared to treatment with either drug alone, regardless of LKB1 status in H1299 cells (Fig 5E). Inhibition of phospho-Akt was also similar in EGF-stimulated cells treated with erlotinib in combination with PF-228 regardless of LKB1 status in H1299 (Fig 5F). These results suggest that the increased sensitivity of A549 cells compared to the other parental cell lines tested in our initial experiments is not due to the genetic loss of LKB1 in parental A549 cells.

### Combination treatment with erlotinib and FAK TKI effectively decreases 3-dimensional growth of lung tumor cells in vitro and tumor growth in vivo

**Fig 6:**
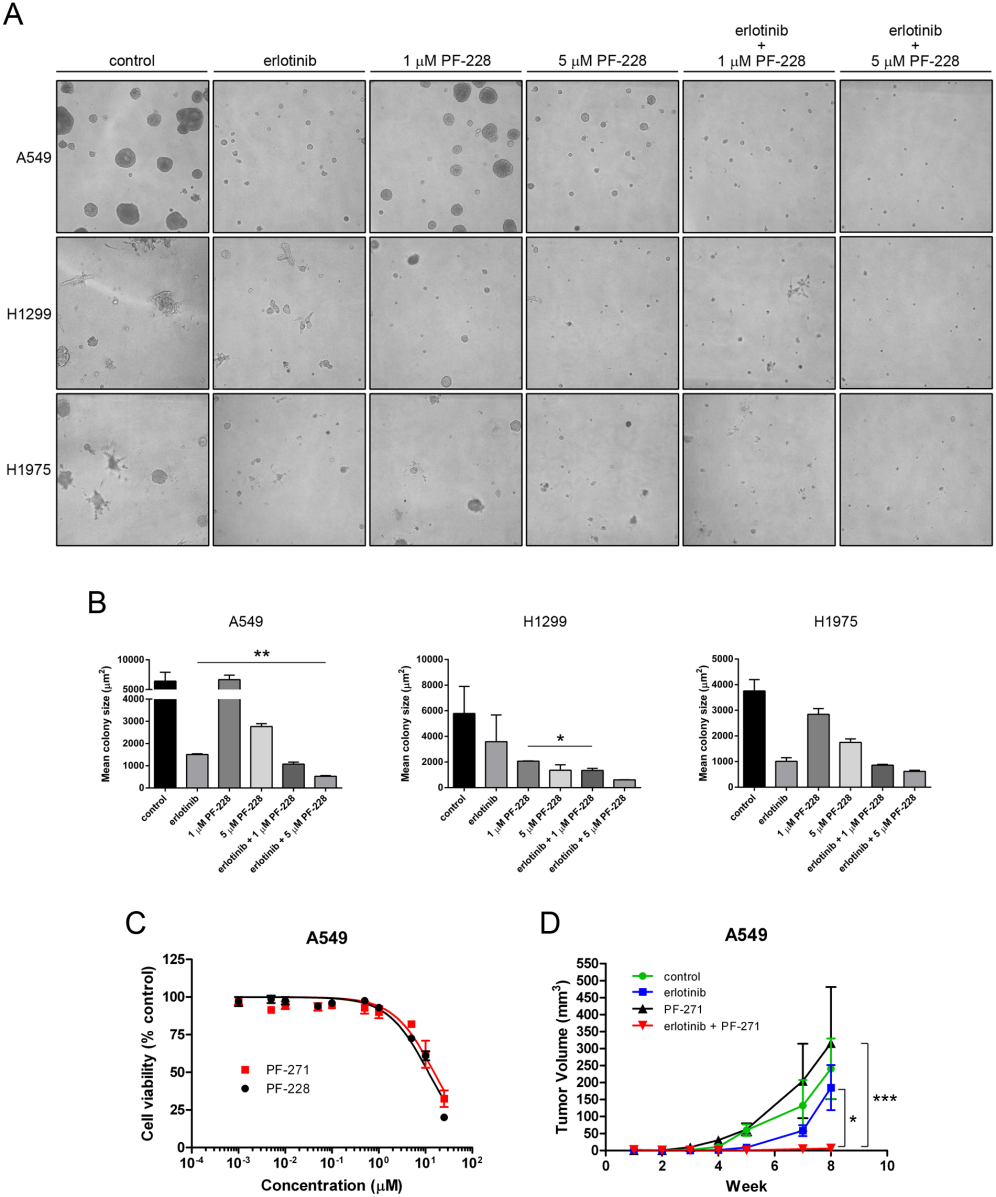
Combination treatment with erlotinib and FAK inhibitor is more effective in reducing colony growth *in vitro* and tumor growth *in vivo*. (**A**) 3-dimensional colony formation was assessed from NSCLC cells embedded in growth factor reduced BME in 4-well chamber slides in the presence of either vehicle control (DMSO), erlotinib (10 μM), PF-228 (1 μM or 5 μM), or the combinations of erlotinib and PF-228 in complete media. Media with fresh drug was replenished every 3 days and colony size was assessed after 13 days. Images representative of two independently performed experiments are shown. (**B**) Mean colony area ± SEM is presented with statistically significant differences in mean colony size determined by unpaired Student’s *t* tests (** *P* = 0.0025, A549; * *P* = 0.044, H1299) from two independently performed experiments. (**C**) FAK inhibitors PF-228 and PF-271 show similar effectiveness in reducing cell viability *in vitro*. A549 cells were treated with either inhibitor for 48 hours and cell viability was assessed by MTT assay. Data indicates the mean ± SEM for log[inhibitor] vs. normalized response from two independently performed experiments. (**D**) *In vivo* data was collected from CD-1 nude mice injected subcutaneously with A549 cells and treated by oral gavage beginning 3 days post tumor cell injection. Data presented is the mean ± SEM for tumor volume from 4 mice per treatment each with cells injected into both hind flanks (n = 8 tumors/treatment), with statistically significant differences in tumor volume at week 8 determined by ANOVA (* P < 0.05, *** P < 0.001).

To assess the ability of FAK blockade to inhibit the colony growth of lung cancer cells in 3-dimensions, a setting that better mimics the potential growth of these cells *in vivo*, NSCLC cells were embedded in growth factor-reduced BME in complete growth medium and treated with either erlotinib (10μM), PF-228 (either 1 or 5 μM), or the combination of both erlotinib and PF-228. Colony formation by A549, H1299 or H1975 cells was assessed from two independently performed experiments with an evident difference in colony size between treatment groups seen after 13 days in all three cell lines tested (Fig 6A). While the number of colonies appeared similar in all treatment groups, a shift in the distribution of colony size towards smaller colonies was observed for both erlotinib treated and PF-228 treated cells (Fig 6A and 6B). Importantly, significant decreases in colony size were observed in A549 and H1299 cells treated with the combination of erlotinib and PF-228 compared to either treatment alone (Fig 6B; A549, *P* = 0.0025 vs. erlotinib; H1299, *P* = 0.044 vs. erlotinib), again indicating the efficacy of combining these two treatments. Although an evident decrease in colony size existed in H1975 cells treated with the combination of erlotinib and 5 μM PF-228 as compared to erlotinib treatment alone, this result was not statistically significant. Given that colony number is not affected but colony size is affected by treatment with FAK TKI in combination with erlotinib, our data supports the notion that the combination treatment can also block 3D growth and proliferation of NSCLC cells, again with the most pronounced effects in A549 cells. This suggested it may also have demonstrated efficacy *in vivo*.

As A549 cell growth in 3D appeared to be most significantly affected by the combination treatment, we tested the effectiveness of a combination treatment of erlotinib and FAK inhibitor *in vivo*, following injection of 1 x 10^6^ A549 lung tumor cells into both hind flanks of CD-1 nude mice. Animals were subsequently treated with vehicle control (5% gelucire in PBS), erlotinib, FAK inhibitor PF-271 [23], or the combination of both drugs by oral gavage daily. We chose to use PF-271 for the *in vivo* experiments as it is orally bioavailable and more closely related to the FAK TKIs currently being tested clinically. We could also show that the sensitivity of A549 cells to PF-228 and PF-271 in 2-dimensional growth assays *in vitro* was identical (Fig 6C). Growth of A549-derived tumors over time indicated that treatment with erlotinib alone resulted in a slight but insignificant reduction in tumor growth while treatment with PF-271 alone had no effect on tumor growth *in vivo* at the dosing regimen used (Fig 6D). In contrast, treatment with the combination of PF-271 and erlotinib had a drastic effect on A549 tumor growth *in vivo*, with tumors being significantly smaller than tumors treated with either erlotinib (*P* < 0.05) or PF-271 (*P* < 0.001) alone (Fig 6D).

## Discussion

The limited effectiveness of EGFR TKI treatment for EGFR wild-type NSCLC is established [9,10], thus necessitating the identification of additional signalling pathways that can be targeted for therapeutic intervention. Expression of the non-receptor tyrosine kinase FAK has been shown to correlate with aggressive disease stage in NSCLC [27] and inhibitors of FAK are effective at inhibiting tumor growth in various mouse xenograft models [23,24]. Recently, more attention has focused on the use of FAK inhibitors in combination with existing therapeutic compounds to enhance efficacy [24–26]. In NSCLC cells resistant to the TKI dasatinib, the addition of FAK inhibitor PF-271 to dasatinib treatment inhibited tumor growth to a greater extent than either treatment alone, indicating the contribution of FAK to dasatinib resistance in NSCLC [44]. These data support our findings that blockade of FAK may be central to enhancing response to various targeted therapies. It has been well established that integrin-FAK mediated crosstalk can regulate signalling events within cells in the absence of ligand stimulation of EGFR [45,46], which could contribute in part to a lack of response to EGFR kinase blockade. In comparison to cell lines HCC827 and HCC4006 which harbour the EGFR deletion in exon 19 and have documented sensitivity to EGFR TKIs [34], EGFR wild-type cell lines A549 and H1299, and H1975 cells with the T790M mutation conferring acquired resistance to EGFR TKIs were confirmed in our study to be relatively insensitive to erlotinib, as would be expected based on previous studies [34,37–39]. When these cell lines were treated with the FAK inhibitor PF-228, the erlotinib insensitive EGFR wild-type cell lines proved more sensitive than the cell lines carrying EGFR TKI-sensitizing mutations. Interestingly, the H1975 cell line carrying the T790M acquired resistance mutation, along with the generated HCC4006 with acquired erlotinib resistance were slightly less sensitive to FAK TKI treatment alone than either EGFR wild-type cell line. Taken together, these results suggest that EGFR wild-type, not EGFR mutant NSCLC with inherent resistance to EGFR inhibitors as opposed to acquired resistance, are more likely to show effective tumor inhibition using this combination treatment. The reasons for this are at present unclear, however may reflect the possibility that in the presence of these EGFR gain of function mutations, or development of acquired resistance to EGFR TKIs, FAK activity may play less of a role in enhanced NSCLC cell survival. Generally, however, our data supports the contention whereby addition of a FAK TKI to treatment with erlotinib resulted in enhanced sensitivity of known erlotinib insensitive cells to this agent. Our data suggests that the use of FAK TKIs in combination with erlotinib affects tumor cell growth in both 2D and 3D, in conjunction with increased induction of apoptosis. While more modest induction of apoptosis was observed in H1975, and H1299 cells, apoptosis was increased by 6-fold in A549 cells treated with FAK TKI and erlotinib combinations. This translated into a more efficacious block of A549 tumor growth *in vivo* with the drug combination than was observed following treatment with either drug alone.

In addition to increased apoptosis with the use of the drug combination, treatment with FAK TKI and erlotinib in combination also resulted in significant inhibition of phospho-Akt in A549 cells. Recent evidence has directly linked integrin expression to EGFR TKI resistance in NSCLC cell lines with reduced phosphorylation of ERK and Akt contributing to increased sensitivity to gefitinib in cell lines depleted of β1 integrin [28]. FAK is known to promote cell survival through enhancing the activity of Akt [47], and persistent Akt activation is associated with a lack of response to EGFR TKIs [40], thus making regulation of Akt an important likely contributor to the decreased cell viability observed upon treatment with erlotinib and FAK TKIs. Our data supports this notion, whereby the drug combination resulted in the most inhibition of cell viability and increased apoptosis concomitant with the highest reductions in Akt activity. This is also supported by the fact that we observed the most potent inhibition of phospho-Akt in A549 cells where we also observed the most significant induction of apoptosis and potent inhibition of *in vivo* tumor growth. Similar effects on Akt phosphorylation have been observed following combination treatments of EGFR TKI-resistant cell lines with gefitinib and the mTOR inhibitor everolimus [48], indicating that reduction of Akt activity may be a common identifier of effective drug combinations for EGFR TKI-resistant cells.

Treatment of NSCLC cells with FAK inhibitor alone or in combination also reduced the growth of NSCLC cells in 3-dimensional assays, with the combination treatment being more potent. When tested *in vivo*, the combination of erlotinib and the FAK inhibitor PF-271 was also more potent than either treatment alone in the A549-derived tumor xenograft model. Similar inhibition of A549-derived tumor growth has been observed with cationic liposome-delivered shRNAs targeting FAK and EGFR with reduced microvessel density being observed in treated tumors [49]. As inhibition of the angiogenic activities of endothelial cells has been observed following administration of FAK inhibitors [20], it is possible that the anti-angiogenic effects associated with FAK inhibition may also contribute to the potent inhibition in tumor growth we observed following treatment with both erlotinib and PF-271.

The reason why A549 cells appear more sensitive to the drug combination than the other two EGFR TKI resistant cell lines tested in this current study remains unclear; however, genetic differences are known to exist between A549, H1299, and H1975 cells that may account for their differing responses to FAK inhibition. A549 and H1299 cells are EGFR wild-type while H1975 cells have both the L858R mutation, resulting in increased sensitivity to EGFR TKIs [50], in addition to the T790M mutation responsible in the majority of patients for acquired resistance to EGFR TKIs [50,51]. Additionally, A549 cells harbour a mutation in the LKB1/STK11 gene resulting in reduced LKB1 expression while H1975 and H1299 cells do not contain this mutation [41,42]. As LKB1 has recently been shown to interact with and inhibit FAK [43], and ~30% of lung adenocarcinomas harbour an inactive LKB1/STK11 gene [41], we investigated whether A549 cells may be more sensitive to FAK inhibitors owing to an increased reliance on FAK signalling due to their lack of a LKB1-FAK inhibitory interaction. Our results suggest that similar sensitivities to erlotinib in combination with FAK inhibitors, along with similar efficacy in suppression of phospho-Akt following combination drug treatment are observed regardless of LKB1 status, as demonstrated following both overexpression of LKB1 in A549 cells harbouring mutant LKB1 and in depletion of LKB1 in H1299 cells with wild-type LKB1. Given that LKB1 does not appear to confer the enhanced sensitivity to the drug combination observed in A549 cells, other genetic differences between A549 and H1299 cells require additional focus when determining the effectiveness of FAK inhibitor treatment in NSCLCs with different genetic backgrounds. One potential A549 mutation which may play a role is KRAS, which has been suggested to result in increased FAK activity in NSCLC downstream of RhoA activation [52], and is the topic of ongoing investigations.

Our findings demonstrate that inhibiting FAK is an effective means of sensitizing EGFR wild-type NSCLC cell lines to EGFR TKIs and that FAK inhibitors used in combination with erlotinib may be more effective than treatment with erlotinib alone in inhibiting EGFR TKI-resistant NSCLC cell viability and tumor growth. These results support further investigation into the inclusion of a FAK TKI to treatment with EGFR TKIs such as erlotinib, particularly in NSCLC with wild-type EGFR and inherent erlotinib resistance. Moreover, the context of efficacy in different genetic mutation backgrounds warrants further examination, given the increased efficacy noted in A549 cells compared to the other cells tested in our study.

## Abbreviations

DMSO: dimethyl sulfoxide
ECM: extracellular matrix
EGFR: epidermal growth factor receptor
FAK: focal adhesion kinase
LKB1: liver kinase B1
NSCLC: non-small cell lung cancer
PBS: phosphate buffered saline
PF-228: PF-573,228
PF-271: PF-562,271
TKI: tyrosine kinase inhibitor
